# How Do Firms Promote Green Innovation through International Mergers and Acquisitions: The Moderating Role of Green Image and Green Subsidy

**DOI:** 10.3390/ijerph18147333

**Published:** 2021-07-08

**Authors:** Hang Wu, Yiying Qu

**Affiliations:** Business School, East China University of Political Science and Law, Shanghai 200042, China; wuhang0503@163.com

**Keywords:** green innovation, exploratory international M&As, exploitative international M&As, green image, green subsidy

## Abstract

Carrying out green innovation is beneficial for firms to cope with environmental protection pressure and achieve sustainable development. Existing research has paid enough attention to the driver of green innovation, but still does not reveal how green innovation can be conceived and realized. This paper answers the above question from the perspective of international M&As, examines the relationship between exploratory international M&As, exploitative international M&As and green innovation performance, and further probes into how strategic and environmental factors moderate the green innovation effectiveness of exploratory and exploitative M&As. Results indicate that exploratory and exploitative international M&As both are beneficial for green innovation performance, and to maximize green innovation performance, implementing exploratory M&As is more beneficial for firms pursuing high green image and operating in a high green subsidy environment. By contrast, carrying out exploitative M&As is more effective for firms pursuing low green image and operating in a low green subsidy environment. Theoretical and managerial implications are discussed.

## 1. Introduction

Due to decades of extensive economic growth mode, the global manufacturing firms have brought serious resource consumption and environmental destruction while maintaining rapid growth. What followed is that manufacturing firms begin to be strictly monitored and criticized by multiple stakeholders for their wrongdoing, such as release of toxic materials and emissions that augment global warming [1]. The government has formulated strict environmental protection policies that impose cost on serious pollution emissions, and consumers also tend to take environmental philosophy into consideration when purchasing products and services [2]. Under such circumstances, green innovation has emerged as a sensible measure for firms to respond to these mounting pressures and achieve sustainable development. Empirical research has also confirmed that green innovation can enable firms to gain competitive advantage, financial performance, and environmental performance [3].

Previous studies have explored the antecedents of green innovation from the perspective of technology push, market pull, and regulation push/pull that highlights the vital role of external market and regulatory factors and internal techno-organizational capabilities in driving green innovation [1,2,4,5]. While these studies help us understand why some firms are more willing or capable to implement green innovation, little is still known about how to implement green innovation especially when firms generally lack the corresponding resources and capabilities. In other words, existing research explores the driver of green innovation with a “mechanistic stimulus-response approach” and assumes that firms have the resources and capabilities to implement when pushed or pulled by market or regulatory factors [6]. However, some researchers have argued that green innovation is more complicated than other innovations, and force firms to seek different and heterogeneous knowledge inputs from external sources [7]. Evidence has been found in the literature on the relationship between R&D cooperation, external knowledge sourcing, and green innovation [6,8]. What is worth mentioning is that this group of research based on the perspective of networking or external knowledge search has just started, lacking research from an international perspective. Theoretical research has confirmed that international M&As, referring to international mergers and acquisitions, is an important resource acquisition channel that can provide firms with complementary technology and knowledge needed for innovation [9]. Therefore, studying the relationship between international M&As and green innovation has important theoretical and practical implications.

**Figure 1:**
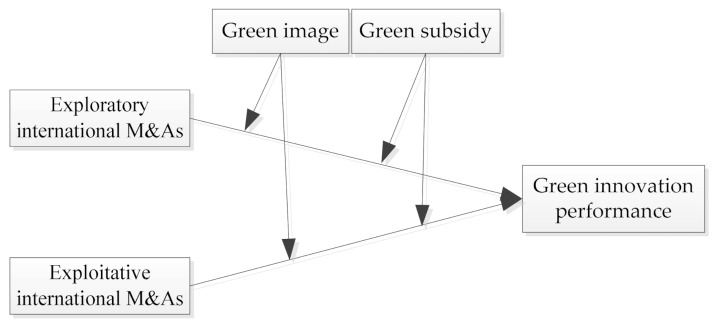
Theoretical model.

Against this background, this paper tries to answer the question of how green innovation can be conceived and realized from the aspect of international Mergers and Acquisitions (M&As), and empirically tests the impact of different international M&As strategies on green innovation. Theoretical research has divided international M&As into two types of exploration and exploitation, and emphasized the necessity of implementing both strategies [10,11,12], to date, however, a few, if any, empirical studies have examined the innovation consequences and moderating conditions of exploratory and exploitative M&As overseas. We propose that exploratory and exploitative international M&As both are beneficial for green innovation. Exploratory international M&As can provide firms with novel and new knowledge beneficial for eco-design and process enhancement, while exploitative international M&As can help firms find the most cost-effective ways to develop green products. Except that, we argue that the effect of international M&As on green innovation can be best understood by focusing on different international M&As behaviors and the strategic, environmental context facing an organization. When firms pursue high green image and operate in a high green subsidy environment, they will choose to explore in foreign markets and obtain high green innovation performance. In contrast, when firms pursue low green image and operate in a low green subsidy environment, they will choose to exploit through international M&As and gain low green innovation performance. Through this richer explanation and empirical assessment, we contribute to a greater clarity and better understanding of how to implement green innovation and, perhaps more important, under what conditions firms pursue this type of innovation. Figure 1 presents our theoretical model of exploratory and exploitative M&As, and green innovation.

## 2. Theory and Hypotheses

### 2.1. The Driver of Green Innovation

The concept of green innovation has been defined as the introduction of new or modified products, processes, or practices to avoid or reduce environmental harms or achieve ecologically specified sustainability targets [13,14]. It concerns all the changes of ecological design, waste utilization, emission reduction, recycling, and elimination of use of hazardous substances in the production process. Green innovation has a “double externality effect,” including positive technology spillover externality and positive environmental protection externality. Similar to conventional innovation, firms focusing on green innovation cannot obtain all the benefits from R&D investment, resulting in a weakening of the firm’s innovation intention. Unlike the conventional innovation, the environmental protection externality makes the R&D benefits of green innovation occupied by the public, which is manifested by the reduction of pollution emissions and the continuous improvement of the environment.

Because of market-failure derived by the interaction of the above two externalities, existing studies have pointed out that firms are not willing to conduct green innovation, which is affected by external environment such as government regulations and stakeholder requirements. Regarding government regulations, existing research has confirmed that the government can push and pull firms to conduct green innovation through strict environmental protection policies and encouraging public subsidies [2,5]. As for stakeholders, the extant research has demonstrated the central role of customer, supplier, competitors in green innovation. Peng and Lin [15] argue that local stakeholders exert tremendous isomorphic pressure on subsidiaries to carry out green innovation. Lin et al. [16] propose that customers’ green requirements compel firms to produce sustainable products and integrate environmental-friendly production processes. Except that, recent research shows that green innovation also requires firms to own certain technological and organizational capabilities, including R&D and financial capability [2], environmental management systems [5], QMS certifications [2].

Compared with the above research on technology push, market pull and regulatory push/pull effects, the extant literature has instead explored marginally the drivers of green innovation that work through the interaction between the firm and its external environment [6], especially from the perspective of international M&As. Among the few recent studies, researchers have shown that firms should search outside the boundary from customers, suppliers, and research institutions to obtain complementary resources because green innovation is more complicated than conventional innovation [6,8,17]. Carrillo-Hermosilla et al. [18] even directly point out that product design, user participation, efficiency improvement, and other issues in the green product and process innovation require a lot of novel knowledge and knowledge interactions, which ask the firm to be more open and search in foreign markets. As a further step toward substantiation of this view, it is necessary to investigate whether and under what conditions international M&As is beneficial for green innovation. The research is of great significance for companies and government departments to design green innovation policy tools.

### 2.2. The Nature of Exploratory and Exploitative International M&As

Traditional international business literature generally views international M&As as a channel for firms to transfer and exploit firm-specific competitive advantages overseas [19]. Recent studies have recognized that firms investing in foreign countries can not only exploit own advantages but also explore and capture new and necessary resources to strengthen corporate long-term competitiveness [10,20]. Therefore, companies will engage in international M&As by the process of either exploration or exploitation. However, previous research on the relationship between international M&As and innovation has tended to take international M&As as a whole unit of analysis [21] and obtained positive [22,23], negative [24], nonsignificant [25], or even U-shaped [26] empirical results, thereby ignoring the fact that international M&As strategy can be divided into exploratory and exploitative activities and the innovation implications of international behaviors might need to fit with contingency factors.

Building on the organizational learning perspective, March [27] argues that exploration refers to activities such as search, variation, experimentation, and discovery, which can assist in gaining new knowledge and information and improving future returns, whereas exploitation involves activities such as refinement, efficiency, selection, and implementation, which helps the firm use existing knowledge and information and thus increase present returns. Exploration and exploitation involve different aspects of organizational learning. More specifically, exploration represents learning gained through distant and shallow search for unknown knowledge along an entirely different trajectory, while exploitation represents learning gained through local search and repeated use for known knowledge along the same trajectory [28,29]. In view of this, explorative international M&As can be defined as a kind of learning activity through broad search for new knowledge and complementary assets in the M&As process, which are far beyond the current experience of the firm. By contrast, exploitative international M&As refer to learning activity through local and deep search for repeated usage of the same knowledge in the M&As process, which relates to the firm’s previous experience and knowledge domain.

### 2.3. Impacts of Exploratory and Exploitative International M&As on Green Innovation Performance

#### 2.3.1. Exploratory International M&As and Green Innovation Performance

Compared to the traditional innovation, green innovation is characterized by higher levels of novelty, uncertainty, and variety, which usually needs to be supported by novel, complex and sophisticated knowledge outside their technological or market domain [30]. Exploratory international M&As arises out of a necessity for firms to learn and obtain novel technological and marketing knowledge and information in overseas markets. Exploratory international M&As can positively influence green innovation in two aspects [29,31]. On the one hand, exploring overseas can enrich firms’ knowledge pool by adding new variants of technological and market information. The rise in the amount and novelty of knowledge repository may increase international firms’ innovative problem-solving capability during the process of green product design, green process reorganization, and resources deployment for a sustainably better way. On the other hand, exploring overseas can provide firms with new insights and innovative ideas which help firms differentiate from other competitors. As the knowledge involved in green innovation is new and represents a technological frontier, which is usually ignorant to the firm itself and domestic peers, so exploring overseas can help improve the success rate of green innovation and win the market. Mihalache et al. [32] argues that novel knowledge sourced overseas can help firms increase useful knowledge recombination, update the thinking mode, and win out over competitors. Therefore, we propose that:

**Hypothesis** **1.** *Exploratory international M&As is positively related to green innovation performance.
*

#### 2.3.2. Exploitative International M&As and Green Innovation Performance

The aim of conducting exploitative activities overseas for international firms is to fully use their competitive advantages in foreign markets. Exploitative international M&As can positively influence green innovation in two ways [29,31]. First, repeatedly using the firm’s technological and marketing knowledge elements can help firms find the most cost-effective and green ways to develop green products, which is necessary for quickly responding to market demand and government requirements. After many times of production including material supply, personnel allocation and error troubleshooting, green firms know all about internal product design and production process, and just need to integrate green ideas into existing products and redesign the product production process [4]. Exploitative international M&As can help firms reduce errors in problem-solving and avoid mistakes in green product development process. Second, increased familiarities with an existing operational domain through exploitative international M&As provides firms with greater opportunities to find new ways for the recombination of existing knowledge, which helps gain new insight into the green innovation process. Through exploiting overseas again and again, firms can redesign the process and green the supplier to improve resource use efficiency, reduce wastes and energy consumption [33]. Therefore, we propose that:

**Hypothesis** **2.** *Exploitative international M&As is positively related to green innovation performance.
*

### 2.4. Strategic and Environmental Contingencies of Exploratory and Exploitative International M&As

We argue that a firm’s green innovation performance can be both positively affected by the choices of exploration and exploitation strategies in its international M&As, but even more importantly, the green innovative effects of exploratory and exploitative international M&As are subject to a joint consideration of their fit with internal strategic and external environmental factors. Contingency scholars have argued that the best way to organize depends on the nature of the environment to which the organization must relate [34], and decision-maker needs to work hard to achieve the alignment between organizational goal and the internal and external environmental conditions [35]. Therefore, we can imagine that the green innovation performance of international firms depends on the congruence between the choices of exploratory and exploitative international M&As and the environment and strategy.

#### 2.4.1. Strategic Fit: Exploratory International M&As, Exploitative International M&As and Green Image

Corporate image refers to the desired general impression and evaluation of the firm in the minds of its key stakeholders, which can be established and adjusted through public relations and green innovation activities [36]. The establishment of green image is based on achieving the goal of sustainable development, with an emphasis on environmental protection and environmental commitments. A firm with high green image will pay great attention to environmental concerns and social benefits, focus on fulfilling corporate social responsibility, and invest a lot of resources for firm’s long-term development [3]. Existing research has proved that investment in getting high green image can help enhance consumer satisfaction and increase sales of green products and service, avoid the potential trouble of environmental protests and legal penalties [37], which in turn enables firms to be more willing and capable to invest in related activities for realizing green innovation. This means that when firms pursue high green image, they are scheduled to guide resources to invest in conducting exploratory international M&As and leveraging the knowledge sourced for green innovation. In contrast, at this time, exploitative international M&As activities will not be supported by sufficient resources, which in turn will also result in the inability of firms to achieve good green innovation performance. However, when firms pursue low green image, they will guide resources to invest in general innovation related activities, then the implementation of exploratory international M&As and the transformation of novel knowledge explored overseas into green innovation both are difficult to conduct. In other words, pursuing low green image may hinder exploration-oriented firms from seeking novel knowledge to enhance green innovation. Under this condition, exploitative international M&As can be used to enhance green innovation performance because of its low resource requirements. Therefore, we propose that:

**Hypothesis** **3a.** *When firms pursue high green image, firms will benefit higher green innovation performance from exploratory international M&As.
*

**Hypothesis** **3b.** *When firms pursue low green image, firms will benefit higher green innovation performance from exploitative international M&As.
*

#### 2.4.2. Environmental Fit: Exploratory International M&As, Exploitative International M&As and Green Subsidy

Green subsidy, also known as environmental subsidy, is a kind of an industrial policy provided by the government to protect the environment and natural resources, which is presented as such forms of payment of cash, tax incentives and exemptions, government environmental protection investments or government loans at preferential rates [2]. Due to the increasing environmental damage and “double externality effect,” governments of all countries adopt intervention policies to internalize environmental costs, subsidize the investment of domestic companies in governing the environment, and improving product processing technology and environmental protection equipment [38]. High green subsidy means firms can obtain a lot of financial support to conduct international M&As and seek enough novel knowledge for green innovation [3]. However, we argue that firms tend to explore foreign markets to boost green innovation when the government provides high green subsidy, as exploration can offer firms new, novel, and sophisticated knowledge for green innovation. High green subsidy can also enable international firms convert knowledge explored overseas into green innovation performance. In contrast, firms will just obtain average green benefits from foreign exploitation activities in a high green subsidy environment, because exploitation takes place in familiar knowledge areas which cannot play such a big role as exploratory international M&As in green innovation. It is worth noting that although exploratory international M&As provide firms with a lot of novel knowledge and information, it is a real high-risk challenge for them to devote or borrow enough resources to integrate the knowledge learned overseas. Therefore, when firms get low green subsidy in implementing green innovation, it is difficult for firms to explore overseas and the innovation effect of exploratory international M&As may be really limited, then it is wise for firms to conduct exploitative international M&As with low risk and resource investment, which enable them to make more efficient use of their existing resources and capabilities. Therefore, we propose that:

**Hypothesis** **4a.** *In a high green subsidy environment, firms will benefit higher green innovation performance from exploratory international M&As.
*

**Hypothesis** **4b.** *In a low green subsidy environment, firms will benefit higher green innovation performance from exploitative international M&As.
*

## 3. Methodology

### 3.1. Sample and Data Collection

We used questionnaire survey to collect data from international manufacturing enterprises located in the industrial parks in Zhejiang province in China. Due to the rapidly developing economy and advanced management philosophy, the Zhejiang provincial government has invested a lot of energy in environmental protection and sustainable development. Under this regulatory condition, firms in Zhejiang province have to pay great attention to green innovation and are forced to participate in international market competition to learn advanced technological knowledge. Thus, the Zhejiang context offers a rich setting to test our hypotheses. We sent questionnaires to 700 firms and asked the senior executives in charge of international operations and green innovation management as the key respondent. The questionnaire is designed according to the mature scale of classic literature, and distributed in two rounds. During the first round of questionnaire distribution, the team members explained the purpose of the survey in detail by phone or email in advance, and promise to mail a copy of the research report to them if they need it. We received 72 valid questionnaires in one month. After the second round of phone, fax, and personal follow-ups, we obtained the other 155 valid questionnaires. In the end, 227 valid questionnaires (32.4%) were returned after deleting replies with missing observations. Of the responding multinational enterprises, enterprises with the largest proportion (30.40%) are located in the electronic information industry, followed by enterprises located in special equipment manufacturing (19.38%), transportation equipment manufacturing (11.89%), ordinary machinery manufacturing (12.78%), metal products industry (9.25%), and others (16.30%). Since there is only one person filling in each questionnaire, it is necessary to test the common method bias. Harmon’s one-factor test showed that no single factor explained a majority of the variance, demonstrating that common method bias was not a serious concern in this study.

### 3.2. Measures

#### 3.2.1. Dependent Variable

Following the approach of Chen et al. [39], Chen [40], and Xie et al. [3], we used six survey items to measure green innovation performance, which reflected the performance conditions in terms of green product innovation and green process innovation. A seven-point Likert scale was used to measure the multi-item variable, asking the respondents to rate the extent that the firm has made changes in product innovation and process innovation relating to energy-saving, pollution-prevention, waste recycling, no toxicity, or green product designs.

#### 3.2.2. Independent Variables

The measurement scales of exploratory and exploitative international M&As were developed based on the understanding of Makino et al. [10] and Hsu et al. [20], with the measurement items adapted from Cui, Meyer and Hu [41] on strategic asset seeking, market seeking and efficiency seeking, adjusted from Luo [42] on capability building and capability exploitation. Exploratory international M&As was measured by five seven-point Likert scale items reflecting the extent to which their firms have sought high-level R&D and management talent and technological and marketing resources through international M&As. Exploitative international M&As was measured by five items that asked respondents to indicate the extent to which their firms have leveraged technological advantages in foreign markets for market expansion or cost reduction purpose. The specific measurement items can be seen in Table 1.

#### 3.2.3. Moderating Variables

Based on the work of Amores-Salvadó et al. [36] and Xie et al. [3], a three-item scale was used for green image tapping into the firm’s awareness of environmental risks and environmental achievements, asking the respondents to assess the degree that firms (1) have raised awareness about the environmental risks and impacts, (2) have been regarded as the best benchmark of environmental management, (3) have demonstrated the ability to reduce waste via corresponding environmental performance. Following the understanding and measurement of Xie et al. [3], green subsidy was measured with three items that reflected the extent that firms have received strong financial support from the government related to environmental protection, asking the respondents to assess the degree that (1) firms have obtained a large amount of subsidies related to environmental protection, (2) the government gives a lot of subsidies for environmental protection, (3) the government’s environmental subsidies are encouraging for firms.

#### 3.2.4. Control Variables

Five variables that may affect firm’s green innovation performance were controlled. First, firm size was operationalized as the natural log value of the total employees [43], because large firms tend to have more resources and external network to support green innovation. Second, firm age was operationalized as the number of years since the firm was founded [44], since elder firms always have established stable networks with local government and financial institutions to obtain resources for green innovation. Third, R&D intensity was measured as R&D expenditures as a percentage of sales because firms with higher level of R&D investment tended to have better green innovation performance [45]. Fourth, international experience was measured by the number of years that a firm had engaged in international business [45], as firms with rich international experience are more capable to seek new and novel knowledge overseas. Finally, we used five dummy variables, industry dummy 1 (electronic information), industry dummy 2 (special equipment manufacturing), industry dummy 3 (transportation equipment manufacturing), industry dummy 4 (ordinary machinery manufacturing), and industry dummy 5 (metal product) to control for the industry effects.

## 4. Analysis and Results

### 4.1. Validity of Variable Scales

The reliability and validity tests for our measurement items and scales are shown in Table 1. We used the value of Cronbach’s alpha to test the reliability of the construct [46]. The alpha reliabilities of our constructs were greater than the recommended 0.70 (as seen in Table 1), ranging from 0.913 to 0.985 (0.963 for exploratory international M&As, 0.921 for exploitative international M&As, 0.920 for green image, 0.913 for green subsidy, and 0.985 for green innovation performance), which shows good reliability. We used confirmatory factor analysis (CFA) to test the discriminant validity of the construct. An integrated five-factor CFA showed that the model fits the data well (χ2 = 423.783, *p* = 0.000; χ2/df = 2.129, NNFI = 0.951, CFI = 0.962, IFI = 0.962, RMSEA = 0.071). Item loadings were as proposed and significant (*p* < 0.01), as shown in Table 1. To test the discriminant validity, we also calculated the average variance extracted (AVE) for exploratory international M&As, exploitative international M&As, green image, green subsidy and green innovation performance, and found that the AVE by the measure of each factors were larger than the squared correlation of that factor’s measure with all measures of other factors in the model, strongly supporting the discriminant validity [47].

### 4.2. Descriptive Statistics

Table 2 presents the means, maximum/minimum, standard deviations and correlations for the study variables. Green innovation performance is positively correlated with exploratory international M&As (r = 0.399, *p* < 0.01) and exploitative international M&As (r = 0.415, *p* < 0.01), which lays a good foundation for the following regression analysis.

### 4.3. Tests of Hypotheses

We conducted a hierarchical moderated regression analyses for exploratory international M&As, exploitative international M&As, and green innovation performance to test all hypotheses. Hierarchical moderated regression analysis is an appropriate and widely used technique to test the effects of different predictors and interactions on the dependent variable. The results of moderated regression analysis are shown in the Table 3. As the baseline model, model 1 only includes control variables of industry dummy, firm age, firm size, R&D intensity, international experience, and moderating variables of green image, green subsidy. Model 2 introduces the explanatory variables of exploratory international M&As and exploitative international M&As. Model 3 contains the interaction terms of exploratory international M&As and green image, exploitative international M&As and green image. Model 4 contains the interaction terms of exploratory international M&As and green subsidy, exploitative international M&As and green subsidy. Model 5 contains the control variables, moderating variables, explanatory variables, and all the interaction terms of exploratory international M&As and green image, exploitative international M&As and green image, exploratory international M&As and green subsidy, exploitative international M&As and green subsidy. To reduce multicollinearity, we mean-centered the relevant variables before creating the interaction terms. As shown in Table 3, the calculation of variance inflation factors (VIF) showed the maximum value of VIF in the regression models was below three, indicating that multicollinearity was not a serious problem [48].

Model 2 shows that the coefficients for exploratory international M&As (β = 0.281, *p* < 0.001) and exploitative international M&As (β = 0.258, *p* < 0.001) are positive and significant, Hypotheses 1 and 2 are supported. Model 3 shows that the interaction between exploratory international M&As and green image is positive and significant (β = 0.268, *p* < 0.01), Hypothesis 3a is supported. Model 3 also shows that the interaction between exploitative international M&As and green image is negative and significant (β = −0.166, *p* < 0.01), Hypothesis 3b is supported. In model 4, the significantly positive coefficient for the interaction between exploratory international M&As and green subsidy (β = 0.277, *p* < 0.001) reveals Hypothesis 4a is supported. The significant and negative coefficient for the interaction between exploitative international M&As and green subsidy (β = −0.169, *p* < 0.01) reveals Hypothesis 4b is supported.

Otherwise, results of the full model (Model 5) also show that exploratory international M&As (β = 0.294, *p* < 0.001) and exploitative international M&As (β = 0.214, *p* < 0.001) both are significantly and positively related to green innovation performance. Hypotheses 1 and 2 are supported again. Green image positively moderates the relationship between exploratory international M&As and green innovation performance (β = 0.214, *p* < 0.001), negatively moderates the relationship between exploitative international M&As and green innovation performance (β = −0.133, *p* < 0.05). Hypotheses 3a and 3b are supported again. Green subsidy positively moderates the relationship between exploratory international M&As and green innovation performance (β = 0.246, *p* < 0.001), negatively moderates the relationship between exploitative international M&As and green innovation performance (β = −0.131, *p* < 0.05). Hypotheses 4a and 4b are supported again.

**Figure 2:**
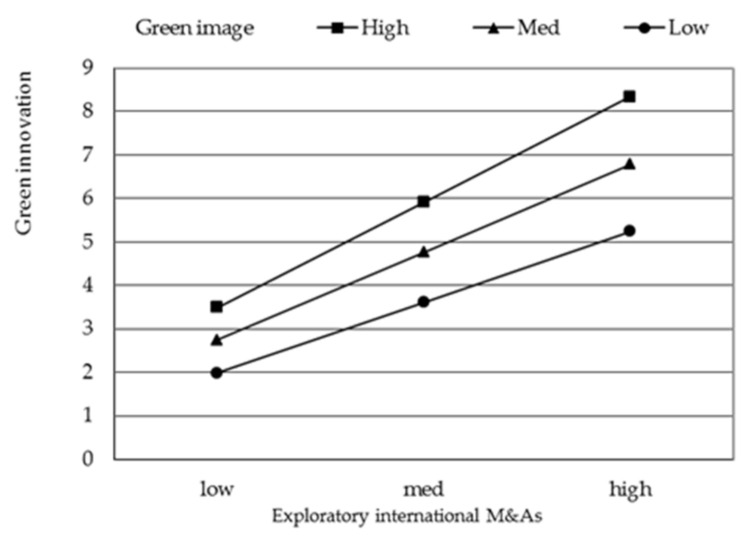
Moderating effect of green image on the relationship between exploratory international M&A and green innovation performance.

**Figure 3:**
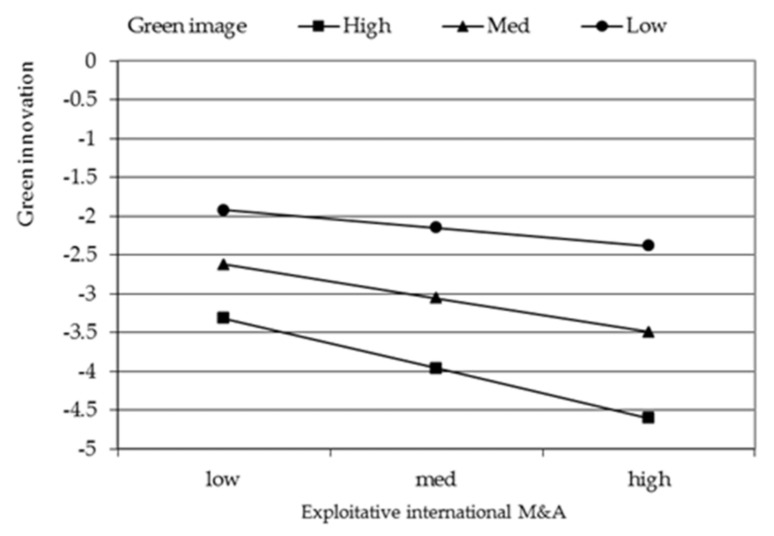
Moderating effect of green image on the relationship between exploitative international M&A and green innovation performance.

**Figure 4:**
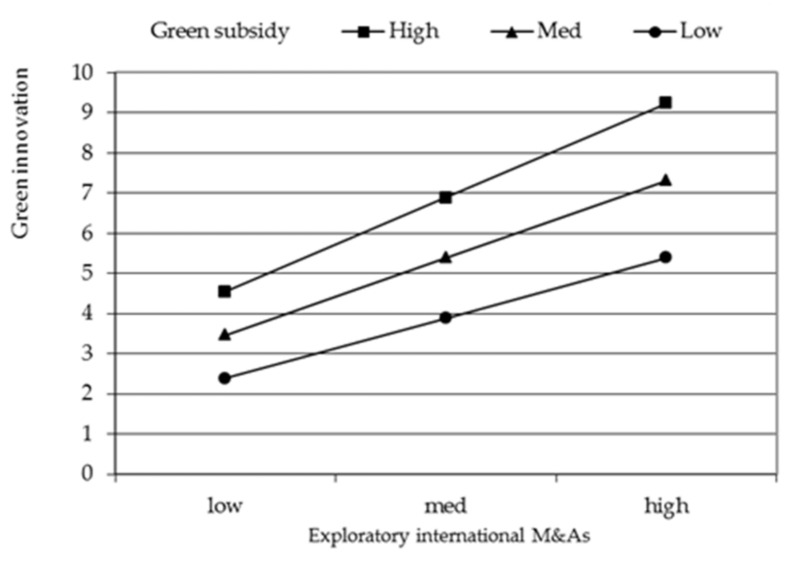
Moderating effect of green subsidy on the relationship between exploratory international M&A and green innovation performance.

**Figure 5:**
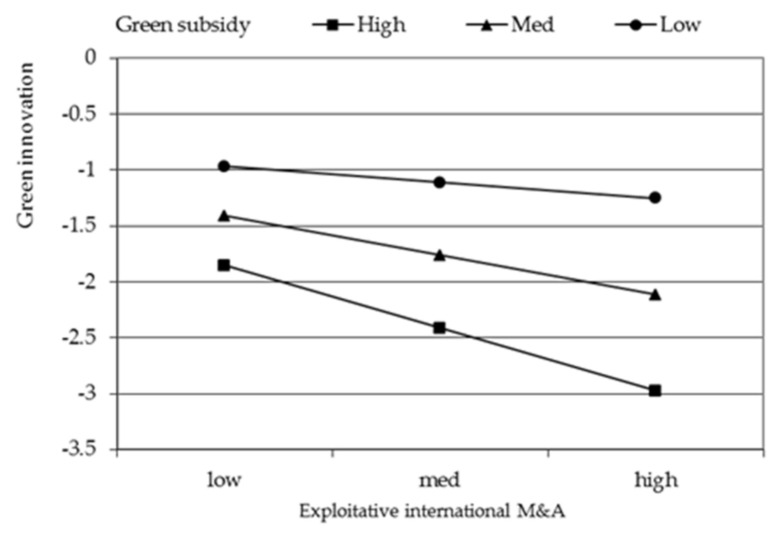
Moderating effect of green subsidy on the relationship between exploitative international M&A and green innovation performance.

To facilitate interpretation, we plot the significant interaction effect of green image and exploratory international M&A in Figure 2, green image and exploitative international M&A in Figure 3, green subsidy and exploratory international M&A in Figure 4, green subsidy and exploitative international M&A in Figure 5 respectively. Exploratory international M&A, exploitative international M&A, green image, and green subsidy all take the values of one standard deviation below and above the mean. As shown in Figure 2, the positive relationship between exploratory international M&A and green innovation performance is stronger when green image is high (one standard deviation above the mean) than when it is low (one standard deviation below the mean). These results thus support hypothesis 2. Figure 3 shows that the positive relationship between exploitative international M&A and green innovation performance is stronger when green image is low than when it is high, supporting hypothesis 3. Figure 4 illustrates that the positive impact of exploratory international M&A on green innovation performance is stronger among firms with a higher green subsidy, supporting hypothesis 4a. Figure 5 illustrates that the positive impact of exploitative international M&A on green innovation performance is stronger among firms with a lower green subsidy, supporting hypothesis 4b.

### 4.4. Supplementary Analyses

One could argue that elder firms may be different from younger firms as firms with older age generally have more experience and problem-solving skills, have established an extensive social network beneficial for international M&As and green innovation. Except that, larger firms may be different from smaller firms as firm size means the number of resources that can be devoted to international M&As and green R&D. So larger firms may have better performance in international M&As and green innovation. To eliminate this concern, we divide our sample into two parts based on firm age and firm size, and re-estimate full model regression analysis for elder firms (n = 113), younger firms (n = 114), larger firms (n = 113), and smaller firms (n = 114). The results remain consistent with our earlier findings, providing additional evidence to the robustness of our findings.

## 5. Discussion

### 5.1. Main Findings

Research on the driver of green innovation is fast-growing in recent years, yet our understanding of how green innovation can be conceived and realized remains rather unclear [49,50]. The objective of this study is to explore the international M&As antecedents of green innovation, and gain a greater understanding of the innovation implication and context conditions of both kinds of international M&As. Our findings demonstrate that the relationship between exploratory and exploitative international M&As and green innovation performance both are positive, empirically confirming firms whether exploring or exploiting overseas can learn helpful knowledge spent on green innovation. The positive green innovation implication of exploratory international M&As is consistent with prior research on exploration and general innovation [51,52], indicating that there do not exist over exploration phenomenon in Chinese international firms. Conversely, the positive argument of exploitative international M&As is contrary to Katila and Ahuja’s [29] inverted U-shaped result on general innovation, testifying that Chinese firm’s exploitation of ownership advantage is still at the initial beginning stage and overexploitation has not yet appeared.

The results of our analyses also reveal the importance of fit between different international behaviors and internal and external context factors in promoting firm’s green innovation performance. Specifically, the results show that firms whose international behaviors fit such internal strategy as pursuing green image will experience higher green innovation performance, meaning that firms pursuing high green image benefit more from exploratory international M&As while firms pursuing low green image benefit more from exploitative international M&As. This finding is consistent with studies in strategic fit literature which emphasizes enterprises’ performance is influenced by the fit or congruency between their internal strategies and international strategy [41]. Similarly, our findings also show that the implementation of exploratory and exploitative international M&As needs to fit with external environment. Firms operating in high green subsidy environment will benefit higher green innovation performance from exploratory international M&As, while firms operating in low subsidy environment will benefit higher innovation performance from exploitative international M&As.

### 5.2. Theoretical and Managerial Implications

The present study makes two important contributions to the literature. On the one hand, our findings can contribute to literature on the driver of green innovation. Although existing research has theoretically and empirically illustrated the driver of green innovation from the aspects of technology push, market pull, and regulatory push/pull, indicating that green innovation is driven by such factors as customer demand, pressure from competitors and shareholders, environmental protection policy and technology [1,2,4,5], fewer studies have responded to the question that how firms can implement green innovation, especially taking into consideration that they lack the knowledge and resources supporting green innovation. Moreover, the extant literature from the perspective of networking and external knowledge search has inspired us that firms dedicated to green innovation can obtain the knowledge and technology necessary from outside, but does not reveal where firms should search [6,8]. Our study contributes to this line of research by empirically demonstrating that firms can search in foreign markets through exploratory and exploitative activities and further uncover under what conditions exploratory and exploitative international M&As are more beneficial for green innovation performance.

On the other hand, our findings also contribute to literature about the relationship between international M&As and conventional innovation in traditional international business literature. Most researchers argue that firms can obtain a lot of resources and learning opportunities through entering into international markets [22,23], but empirical researches on the relationship between international M&As and innovation achieve incompatible conclusion [22,23,24,25,26]. Our study advances the literature of this genre by providing empirical evidence to demonstrate that it is necessary to divide international M&As into exploratory and exploitative types and the innovation effect of both international M&As behaviors depends on their fit with internal strategic and external environmental conditions. 

Our findings also have important practical implications. On the one hand, our results suggest that the international market provides an excellent platform for firms to obtain complementary resources and carry out green innovation. Firms can both conduct exploratory and exploitative activities overseas to seek novel knowledge and enhance green innovation efficiency. On the other hand, our results also inform that firms should notice the fit between international strategy selection and strategic, environmental factors despite of the positive green innovation effect of exploratory and exploitative international M&As. In order to obtain greater green innovation returns, firms pursuing high green image or operating in a high green subsidy environment should implement exploratory international M&As, whereas firms pursuing low green image or operating in a low green subsidy environment should carry out exploitative international M&As.

### 5.3. Limitations and Future Research Directions

Our research still has some limitations which in turn offer opportunities for future research. First, a constraint on the generalizability of the findings in this study is the sample of the Chinese manufacturing industry, and only based on 227 targets which is obviously not large enough, future research would be well served to extend the theoretical model to a cross-country or multi-industry context and collect more firm-level data to test the robustness of the conclusions. Second, this study has just paid attention to the effect of external knowledge through international M&As on green innovation, ignoring to focus on the role played by the internal resources. Therefore, future research can test the comprehensive effect of both internal and external resources on green innovation. Third, we have examined the moderating effects of green image and green subsidy, future research may examine the impact of other moderators at the organization, strategy, and environment levels. For example, such industry-level factors as industry competition and industry growth, and strategy-level factors as cost leadership vs. differentiation, incremental vs. radical innovation strategy, may be rewarding for future research. At last, whereas the present study intentionally focuses on the green innovation consequence of exploratory and exploitative international M&As, an alternative model can consider the ambidextrous effect of two international M&As strategies. Further research should turn to consider the green innovation consequence of international M&As ambidexterity, rather than focusing on the sole use of either exploration or exploitation.

## Figures and Tables

**Table 1:** Construct measurement and confirmatory factor analysis results.

Constructs/Measurement Items	Standardized Loadings
***Exploratory international M&As (Cronbach α = 0.963, AVE = 0.84*** ***0)***	
1. Get high-level R&D and management talent through international M&As	0.895
2. Seek technological and marketing resources for firm’s development	0.897
3. Acquire the managerial know-how for further improvement	0.907
4. Take advantage of advanced R&D infrastructure	0.893
5. Obtain global business information and technology spillover	0.988
***Exploitative international M&As (Cronbach α = 0.921, AVE = 0.70*** ***0)***	
1. Leverage technological advantages in foreign markets	0.848
2. Enter into foreign markets to expand the market space	0.834
3. Intend to lower transportation cost by producing abroad	0.850
4. Utilize cheap labor and raw material in foreign markets	0.827
5. Expand into foreign markets to benefit from investment incentives	0.825
***Green image (Cronbach α = 0.9*** ***20, AVE = 0.7*** ***94)***	
1. Firms have raised awareness about the environmental risks and impacts	0.916
2. Firms have been regarded as the best benchmark of environmental management	0.879
3. Firms have demonstrated the ability to reduce waste via corresponding environmental performance	0.878
***Green subsidy (Cronbach α = 0.91*** ***3, AVE = 0.77*** ***9)***	
1. Firms have obtained a large amount of subsidies related to environmental protection	0.886
2. The government gives a lot of subsidies for environmental protection	0.895
3. The government’s environmental subsidies are encouraging for firms	0.866
***Green innovation performance (Cronbach α = 0.985, AVE = 0.91*** ***6)***	
1.The firm has used an environmentally friendly design and packaging for existing and new products	0.954
2.The firm has reduced the consumption of energy, such as water, electricity, coal, or oil	0.958
3.The firm has reduced the consumption of raw materials	0.952
4.The firm has reduced the emission of hazardous substances or waste	0.953
5.The firm has used raw materials to be easily recycled, reused and decomposed	0.972
6.The firm has used clean technologies and environmental protection equipment to promote energy efficiency and pollution prevention	0.952

Notes: All standardized coefficient loadings are significant at *p* < 0.001; AVE = average variance extracted for each multi-item construct in the research model.

**Table 2:** Means, standard deviations, and correlations.

	Max	Min	Mean	S. D	1	2	3	4	5	6	7	8	9
1.Firm age	42.000	2.000	15.80	8.246	1								
2.Firm size	11.127	4.605	7.536	1.388	0.263 **	1							
3.R&D intensity	0.130	0.010	0.050	0.024	0.052	0.006	1						
4.International experience	30.000	3.000	6.970	3.730	−0.036	0.021	0.457 **	1					
5.Green image	6.670	1.330	4.756	1.232	0.001	−0.163 *	0.019	−0.040	1				
6.Green subsidy	7.000	1.000	4.225	1.251	0.206 **	−0.145 *	0.019	−0.004	0.082	1			
7.Exploratory international M&As	6.600	1.000	3.957	1.349	−0.001	0.198 **	0.220 **	0.172 **	0.017	−0.056	1		
8.Exploitative international M&As	6.800	1.400	4.601	1.059	0.059	0.134 *	0.233 **	0.169 *	0.268 **	0.212 **	0.179 **	1	
9.Green innovation performance	6.833	1.167	3.925	1.659	0.045	0.214 **	0.308 **	0.203 **	0.128	0.146 *	0.399 **	0.415 **	1

Note: ** *p* < 0.01; * *p* < 0.05.

**Table 3:** Results of moderated regression analysis.

	Model 1	Model 2	Model 3	Model 4	Model 5
Industry dummy 1 (electronic information)	−0.022	−0.045	−0.063	−0.085	−0.095
Industry dummy 2 (special equipment manufacturing)	0.060	−0.004	−0.070	−0.058	−0.106
Industry dummy 3 (transportation equipment manufacturing)	−0.002	−0.054	−0.075	−0.102	−0.114
Industry dummy 4 (ordinary machinery manufacturing)	0.048	0.015	−0.011	−0.013	−0.032
Industry dummy 5 (metal product)	0.043	0.007	−0.052	−0.024	−0.069
Firm size	0.300 ***	0.177 **	0.165 **	0.164 **	0.158 **
Firm age	−0.074	−0.046	−0.084	−0.054	−0.085
R&D intensity	0.272 ***	0.177 **	0.152 *	0.106	0.093
International experience	0.072	0.030	0.040	0.068	0.068
Green image	0.139 *	0.054	0.004	0.029	−0.009
Green subsidy	0.184 **	0.141 *	0.139 *	0.159 **	0.156 **
Exploratory international M&As		0.281 ***	0.275 ***	0.303 ***	0.294 ***
Exploitative international M&As		0. 258 ***	0.222 ***	0. 238 ***	0.214 ***
Exploratory international M&As * Green image			0.268 ***		0.214 ***
Exploitative international M&As * Green image			−0.166 **		−0.133 *
Exploratory international M&As * Green subsidy				0.277 ***	0.246 ***
Exploitative international M&As * Green subsidy				−0.169 **	−0.131 *
R^2^	0.210	0.343	0.415	0.438	0.483
F	5.203 ***	8.560 ***	9.995 ***	10.985 ***	11.463 ***
Max VIF	2.521	2.548	2.599	2.588	2.622

Note: Standardized regression coefficients are reported. * *p* < 0.05, ** *p* < 0.01, *** *p* < 0.001.

## Data Availability

The data that support the findings of this study are available from the authors upon reasonable request.

## References

[1] Berrone P., Fosfuri A., Gelabert L., Gomez-Mejia L.R. (2013). Necessity as the mother of ‘green’ inventions: Institutional pressures and environmental innovations. Strateg. Manag. J..

[2] Cuerva M.C., Triguero-Cano Á., Córcoles D. (2014). Drivers of green and non-green innovation: Empirical evidence in Low-Tech SMEs. J. Clean. Prod..

[3] Xie X., Huo J., Zou H. (2019). Green process innovation, green product innovation, and corporate financial performance: A content analysis method. J. Bus. Res..

[4] Albort-Morant G., Leal-Millan A., Cepeda-Carrion G. (2016). The antecedents of green innovation performance: A model of learning and capabilities. J. Bus. Res..

[5] Kesidou E., Demirel P. (2012). On the drivers of eco-innovations: Empirical evidence from the UK. Res. Policy.

[6] Ghisetti C., Marzucchi A., Montresor S. (2015). The open eco-innovation mode. An empirical investigation of eleven European countries. Res. Policy.

[7] Horbach J., Rammer C., Rennings K. (2012). Determinants of eco-innovations by type of environmental impactdthe role of regulatory push/pull, technology push and market pull. Ecol. Econ..

[8] Marchi V.D. (2012). Environmental innovation and R&D cooperation: Empirical evidence from Spanish manufacturing firms. Res. Policy.

[9] Wu H., Chen J. (2020). International ambidexterity in firms’ innovation of multinational enterprises from emerging economies: An investigation of TMT attributes. Balt. J. Manag..

[10] Makino S., Lau C.-M., Yeh R.-S. (2002). Asset-exploitation versus asset-seeking: Implications for location choice of foreign direct investment from newly industrialized economies. J. Int. Bus. Stud..

[11] Prange C., Verdier S. (2011). Dynamic capabilities, internationalization process and performance. J. World Bus..

[12] Bandeira-de-Mello R., Fleury M.T.L., Aveline C.E.S., Gama M.A.B. (2016). Unpacking the ambidexterity implementation process in the internationalization of emerging market multinationals. J. Bus. Res..

[13] Rennings K. (2000). Redefining innovation eco-innovation research and the contribution from ecological economics. Ecol. Econ..

[14] Kemp R. (2010). Eco-innovation: Definition, measurement and open research issues. Econ. Politics.

[15] Peng Y.S., Lin S.S. (2008). Local responsiveness pressure, subsidiary resources, green management adoption and subsidiary’s performance: Evidence from Taiwanese manufactures. J. Bus. Ethics.

[16] Lin R.J., Tan K.H., Geng Y. (2013). Market demand, green product innovation, and firm performance: Evidence from Vietnam motorcycle industry. J. Clean. Prod..

[17] De Marchi V., Grandinetti R. (2013). Knowledge strategies for environmental innovations: The case of Italian manufacturing firms. J. Knowl. Manag..

[18] Carrillo-Hermosilla J., Rio P., Konnola T. (2010). Diversity of eco-innovations: Reflections from selected case studies. J. Clean. Prod..

[19] Dunning J.H. (1981). International Production and the Multinational Enterprises.

[20] Hsu C.-W., Lien Y.-C., Chen H. (2013). International ambidexterity and firm performance in small emerging economies. J. World. Bus..

[21] Martineaua C., Pastoriza D. (2016). International involvement of established SMEs: A systematic review of antecedents, outcomes and moderators. Int. Bus. Rev..

[22] Hitt M.A., Ireland R.D., Harrison J.S., Hoskisson R.E. (1991). Effects of acquisitions on R&D inputs and outputs. Acad. Manag. J..

[23] Vermeulen F., Barkema H. (2001). Learning through acquisitions. Acad. Manag. J..

[24] Hitt M.A., Hoskisson R.E., Johnson R.A., Moesel D.D. (1996). The market for corporate control and firm innovation. Acad. Manag. J..

[25] Healy P.M., Palepu K.G., Ruback R.S. (1992). Does corporate performance improve after mergers?. J. Financ. Econ..

[26] Ahuja G., Riitta K. (2001). Technological acquisitions and the innovation performance of acquiring firms: A longitudinal study. Strateg. Manag. J..

[27] March J. (1991). Exploration and exploitation in organizational learning. Organ. Sci..

[28] Levinthal D.A., March J.G. (1993). The myopia of learning. Strat. Manag. J..

[29] Katila R., Ahuja G. (2002). Something old, something new: A longitudinal study of search behavior and new product introduction. Acad. Manag. J..

[30] Cainelli G., de Marchi V., Grandinetti R. (2015). Does the development of environmental innovation require different resources? Evidence from Spanish manufacturing firms. J. Clean. Prod..

[31] Li C.R., Chu C.P., Lin C.J. (2010). The contingent value of exploratory and exploitative learning for new product development performance. Ind. Mark. Manag..

[32] Mihalache O.R., Jansen J.J.J.P., Van Den Bosch F.A.J., Volberda H.W. (2012). Offshoring and firm innovation: The moderating role of top management team attributes. Strateg. Manag. J..

[33] Lee K.H., Min B. (2015). Green R&D for eco-innovation and its impact on carbon emissions and firm performance. J. Clean. Prod..

[34] Scott W.R. (1992). Organizations: Rational, Natural, and Open Systems.

[35] Venkatraman N. (1989). The concept of fit in strategy research: Toward verbal and statistical correspondence. Acad. Manag. Rev..

[36] Amores-Salvado J., Castro M.D., Navas-Lopez J.E. (2014). Green corporate image: Moderating the connection between environmental product innovation and firm performance. J. Clean. Prod..

[37] Chen Y.S. (2010). The drivers of green brand equity: Green brand image, green satisfaction, and green trust. J. Bus. Ethics.

[38] Xie X., Huo J., Qi G., Zhu K.X. (2016). Green process innovation and financial performance in emerging economies: Moderating effects of absorptive capacity and green subsidies. IEEE Trans. Eng. Manag..

[39] Chen Y.S., Lai S.B., Wen C.T. (2006). The influence of green innovation performance on corporate advantage in Taiwan. J. Bus. Ethics.

[40] Chen Y.-S. (2008). The driver of green innovation and green image—Green core competence. J. Bus. Ethics.

[41] Cui L., Meyer K.E., Hu H.W. (2014). What drives firms’ intent to seek strategic assets by foreign direct investment? A study of emerging economy firms. J. World Bus..

[42] Luo Y. (2002). Capability exploitation and building in a foreign market: Implications for multinational enterprises. Organ. Sci..

[43] Wu H., Liu Y. (2018). Balancing local and international knowledge search for internationalization of emerging economy multinationals: Evidence from China. Chin. Manag. Stud..

[44] Wu H., Chen J., Liu Y. (2017). The impact of OFDI on firm innovation in an emerging country. Int. J. Technol. Manag..

[45] Filatotchev I., Liu X., Buck T., Wright M. (2009). The export orientation and export performance of high-technology SMEs in emerging markets: The effects of knowledge transfer by returnee entrepreneurs. J. Int. Bus. Stud..

[46] Lu Y., Zhou L., Bruton G., Li W. (2010). Capabilities as a mediator linking resources and the international performance of entrepreneurial firms in an emerging economy. J. Int. Bus. Stud..

[47] Li H., Zhang Y. (2007). The role of managers’ political networking and functional experience in new venture performance: Evidence from China’s transition economy. Strateg. Manag. J..

[48] Hoskisson R.E., Hitt M.A., Johnson R.A., Grossman W. (2002). Conflicting voices: The effects of institutional ownership heterogeneity and internal governance on corporate innovation strategies. Acad. Manag. J..

[49] Guo Y., Wang L., Yang Q. (2020). Do corporate environmental ethics influence firms’ green practice? The mediating role of green innovation and the moderating role of personal ties. J. Clean. Prod..

[50] Min Z., Sawang S., Kivits R.A. (2021). Proposing circular economy ecosystem for Chinese SMEs: A systematic review. Int. J. Environ. Res. Public Health.

[51] Atuahene-Gima K., Murray J.Y. (2007). Exploratory and exploitative learning in new product development: A social capital perspective on new technology ventures in China. J. Int. Mark..

[52] Yalcinkaya G., Calantone R.J., Griffith D.A. (2007). An examination of exploration and exploitation capabilities: Implications for product innovation and market performance. J. Int. Mark..

